# Low-coverage whole genome sequencing of eleven species/subspecies in *Dioscorea* sect. *Stenophora* (Dioscoreaceae): comparative plastome analyses, molecular markers development and phylogenetic inference

**DOI:** 10.3389/fpls.2023.1196176

**Published:** 2023-06-06

**Authors:** Ke Hu, Xiao-Qin Sun, Min Chen, Rui-Sen Lu

**Affiliations:** ^1^ Institute of Botany, Jiangsu Province and Chinese Academy of Sciences, Nanjing, China; ^2^ Jiangsu Key Laboratory for the Research and Utilization of Plant Resources, Nanjing, China; ^3^ Jiangsu Provincial Science and Technology Resources Coordination Platform (Agricultural Germplasm Resources) Germplasm Resources Nursery of Medicinal Plants, Nanjing, China

**Keywords:** *Dioscorea* sect. *Stenophora*, plastome, comparative analyses, hotspots, phylogeny

## Abstract

*Dioscorea* sect. *Stenophora* (Dioscoreaceae) comprises about 30 species that are distributed in the temperate and subtropical regions of the Northern Hemisphere. Despite being evolutionarily “primitive” and medically valuable, genomic resources and molecular studies of this section are still scarce. Here, we conducted low-coverage whole genome sequencing of 11 *Stenophora* species/subspecies to retrieve their plastome information (whole plastome characteristics, plastome-divergent hotspots, plastome-derived SSRs, etc.) and polymorphic nuclear SSRs, as well as performed comparative plastome and phylogenetic analyses within this section. The plastomes of *Stenophora* species/subspecies ranged from 153,691 bp (*D. zingiberensis*) to 154,149 bp (*D. biformifolia*) in length, and they all contained the same 114 unique genes. All these plastomes were highly conserved in gene structure, gene order and GC content, although variations at the IR/SC borders contributed to the whole length differences among them. The number of plastome-derived SSRs among *Stenophora* species/subspecies varied from 74 (*D. futschauensis*) to 93 (*D. zingiberensis*), with A/T found to be the most frequent one. Seven highly variable regions and 12 polymorphic nuclear SSRs were identified in this section, thereby providing important information for further taxonomical, phylogenetic and population genetic studies. Phylogenomic analyses based on whole plastome sequences and 80 common protein coding genes strongly supported *D. biformifolia* and *D. banzhuana* constituted the successive sister species to the remaining sampled species, which could be furtherly divided into three clades. Overall, this study provided a new perspective for plastome evolution of *Stenophora*, and proved the role of plastome phylogenomic in improving the phylogenetic resolution in this section. These results also provided an important reference for the protection and utilization of this economically important section.

## 1 Introduction

*Dioscorea* is the largest genus in the family Dioscoreaceae with over 600 species, which contains about ten major clades: *Stenophora*, New World I, New World II, African, Mediterranean, New World III, Compound Leaved, Malagasy, *Shannicorea* and *Enantiophyllum* (Viruel et al., 2016; Couto et al., 2018; Viruel et al., 2018). Among them, *Stenophora*, the subject of our study, coincides with the section *Stenophora* Uline circumscribed by Burkill (1960), and contains about 30 species disjunctively distributed in the Northern Hemisphere (Gao et al., 2008; Vinogradova et al., 2022). The sect. *Stenophora* likely originated in Himalayas-Hengduan Mountains, China, and is the most basal clade of *Dioscorea*, differing from the rest of this genus by having rhizomes, monosulcate pollen and a diploid chromosome number (x = 10) (Wilkin et al., 2005; Gao et al., 2008; Hsu et al., 2013; Viruel et al., 2016; Couto et al., 2018; Noda et al., 2020). Plant species in this section are reported to have great medicinal values. In particular, the rhizomes of *D. nipponica* and *D. zingiberensis* are extensively used to extract diosgenin, which is an important precursor for the synthesis of steroid drugs in the pharmaceutical industry (Gong et al., 2011; Cheng et al., 2021). The immense evolutionary and medicinal value of *Stenophora* species has also brought new challenges to their conservation and sustainable use. One major concern is that the increasing demands for naturally growing plants has threaten their wild populations and genetic variations (Das et al., 2013; Sun et al., 2017). For example, *D. nipponica* has been listed as a secondary-level endangered plant species in China, as its wild resource is facing extinction (Fu, 1992; Chen et al., 2007). Another concern is the misidentification and misuse of *Stenophora* species, as they are similar in morphological characteristics and local names. Therefore, accurate and rapid identification of *Stenophora* species (e.g., molecular markers) is urgently required.

Previous studies of *Stenophora* have concentrated on external morphology, cytology, pollen morphology and phytochemistry (Pei et al., 1979; Huang et al., 2010). Morphological and embryological features have been shown to be important for systematics and species identification of *Stenophora*, and could divide this section into several subclades (e.g., Titova and Torshilova, 2015; Vinogradova et al., 2022), however, it is still difficult to find clear gaps of morphological variations among closely related species (Noda et al., 2020). Moreover, although previous molecular-based studies have accelerated species identification and phylogenetic inference of *Stenophora*, the plastid loci used (e.g., *atpB*, *matK*, *rbcL*) always showed low discriminatory power (Gao et al., 2008; Noda et al., 2020). For example, Gao et al. (2008) revealed that *D. nipponica* was sister to *D. althaeoides*, and *D. nipponica* ssp. *rosthornii* was not related to these two species, but their interspecific relationships receive weak bootstrap support. Evidently more effective molecular markers are needed to solve the remaining phylogenetic dilemma.

Plastomes of land plants generally have a quadripartite circular structure, with a pair of inverted repeats (IRs) separated by a large single-copy (LSC) region and a small single-copy (SSC) region, ranging from 100 to 200 kb in length (Raubeson and Jansen, 2005; Jansen and Ruhlman, 2012; Olejniczak et al., 2016; Lu et al., 2023). Due to many advantages such as highly conserved structure, usually uniparental inheritance, absence of recombination, and large copy numbers, plastome sequences have been widely used for accurate species identification and phylogenetic inferences, especially at low taxonomic levels (Gitzendanner et al., 2018; Yang et al., 2022). Furthermore, comparative plastome genomics could provide essential information for plastome evolution, such as gene loss and IR boundary shifts, and can develop mutational hotspots, which may contribute to species discrimination, phylogenetic, and population genetic studies (Lu et al., 2021; Yang et al., 2022). In sect. *Stenophora*, although some representative plastomes have been sporadically released, previous studies mainly focused on the plastome characteristics of single species (e.g., Wu et al., 2016; Zhou et al., 2016), or performed comparative and phylogenetic analyses only based on a small number of plastomes (e.g., Zhao et al., 2018; Xia et al., 2019). Thus, it is necessary to provide more genomic resources for further understanding the plastome evolution and phylogeny of *Stenophora*.

With the rapid development of next generation sequencing (NGS) technologies, it is cheap and fast to obtain low-coverage (~0.1–10×) of the whole genome sequencing data (or called genome skimming data), which could provide sufficient data for complete plastome assemblies (Straub et al., 2012; Twyford and Ness, 2017; Jin et al., 2020). Besides, the assembled nuclear scaffolds from low-coverage whole genome sequencing data could be used for mining polymorphic nuclear SSRs (nSSRs) (e.g., Liu et al., 2018; Xia et al., 2018; Lu et al., 2022). Here, we performed low-coverage whole genome shotgun sequencing for 11 *Stenophora* species/subspecies (i.e., *D. banzhuana* Pei & Ting, *D. biformifolia* Pei & Ting, *D. collettii* Hook.f., *D. deltoidea* Wall., *D. futschauensis* Uline ex R.Knuth, *D. gracillima* Miq., *D. nipponica* Makino, *D. nipponica* subsp. *rosthorni* (Prain & Burkill) C.T.Ting, *D. spongiosa* J.Q.Xi, M.Mizuno & W.L.Zhao, *D. tokoro* Makino, *D. zingiberensis* C.H.Wright). Using this data, we aimed to i) present the complete and annotated plastome sequences of these 11 *Stenophora* species/subspecies, and assess plastome structural evolution of them; ii) identify plastomic SSRs and mutational hotspot regions (plastome-derived markers); iii) develop polymorphic nSSRs based on assembled nuclear scaffolds of *Stenophora* species/subspecies; and iv) conduct phylogenetic analyses of these species/subspecies using plastome data. Overall, this study will not only provide a valuable resource for species identification and phylogenetic studies of *Stenophora*, but also be useful for conservation and utilization of this economically important section.

## 2 Materials and methods

### 2.1 Plant materials, DNA extraction and genomic sequencing

Fresh leaves of 11 *Stenophora* species/subspecies, i.e., *D. banzhuana*, *D. biformifolia*, *D. collettii*, *D. deltoidea*, *D. futschauensis*, *D. gracillima*, *D. nipponica*, *D. nipponica* subsp. *rosthorni*, *D. spongiosa*, *D. tokoro*, *D. zingiberensis*, were field-collected and dried with silica-gel. The voucher specimens were deposited at Herbarium of Institute of Botany, Jiangsu Province and Chinese Academy of Sciences (NAS) [details about sampling information can be found in Gao et al. (2008)]. For each species/subspecies, genomic DNA was extracted from silica gel-dried leaves using DNAsecure Plant Kit (Tiangen Biotech, Beijing, China), following the manufacturer’s protocol. DNA concentration and integrity were measured by Agilent 2100 BioAnalyzer and agarose gel electrophoresis. Paired-end library with insert size of 350 bp was constructed for each species/subspecies by using the Genomic DNA Sample Prep, and then sequenced on the Illumina HiSeqTM 4000 platform (Illumina, San Diego, California, USA) according to the paired-end 2 × 150 bp protocol. Library construction, genome sequencing and raw data filtering were conducted by Novogene Bioinformatics Technology Co., Ltd., Beijing, China.

### 2.2 Plastome assembly and annotation

After removing library barcodes and filtering low-quality data, the clean reads (about 4 Gb per sample) were used for *de novo* assembly of whole plastome sequences using GetOrganelle v.1.7.6 (Jin et al., 2020), with the default parameters as suggested by its authors. All the assembly graphs were subsequently visually inspected using Bandage v.0.8.1 (Wick et al., 2015). Initial annotations of all newly assembled plastomes were performed with MAFFT v.7 plugin (Katoh and Standley, 2013) in Geneious Prime^®^ 2022.0.1 (https://www.geneious.com) by aligning them to two closely related and previously published plastomes, i.e., *D. aspersa* (NC_039807) and *D. collettii* (NC_037717), and transferring reference annotations to these newly assembled plastomes. Then, the initial annotations were checked and adjusted manually to confirm the accuracy of exon/intron boundaries and start/stop codon locations. All newly generated plastome sequences were deposited in GenBank (accession numbers: OQ525992-OQ526002). High-resolution circular plastome maps of these 11 *Stenophora* species/subspecies were generated using the web-based tool OrganellarGenomeDRAW (OGDRAW) v.1.3.1 (Greiner et al., 2019).

### 2.3 Whole plastome comparison within sect. *Stenophora*

To visualize sequence similarity of plastomes within sect. *Stenophora*, all 11 newly sequenced plastomes and one plastome of *D. villosa* (NC_034686) were aligned using the global alignment program LAGAN (Brudno et al., 2003), and visualized in VISTA browser (Frazer et al., 2004), taking annotations of *D. villosa* plastome as reference. To further illustrate the IR expansions and contractions among *Stenophora* plastomes, the four junctions between two invert repeats (IRs) and large/small single copy (LSC/SSC) regions were identified and compared by Repeat Finder plugin as implemented in Geneious Prime^®^ 2022.0.1 (https://www.geneious.com/plugins/repeat-finder/).

### 2.4 Identification of mutational hotspots and plastome-derived SSRs

To identify mutational hotspot regions for PCR-based identification of *Stenophora* species and subspecies, a total of 12 plastomes (one plastome per species/subspecies, **Table 1**) were first aligned using the MAFFT v.7 plugin (Katoh and Standley, 2013) in Geneious Prime^®^ 2022.0.1. Then, protein coding sequences (CDS), intergenic spacer regions (IGS), introns and tRNAs, with aligned length > 200 bp and the total number of mutations > 0 were extracted from the alignment matrix of these 12 plastome sequences. Finally, the nucleotide diversity (*π*) of these regions was calculated in DnaSP v.6.12.03 (Rozas et al., 2017). In addition, the MISA-web application (Beier et al., 2017) was employed to screen SSRs across the 12 *Stenophora* plastomes, with thresholds (minimum numbers) of 10, 5, 4, 3, 3, and 3 repeat units for mono-, di-, tri-, tetra-, penta-, and hexa-nucleotide SSRs, respectively.

**Table 1:** The basic features of 11 *Stenophora* plastomes newly generated in this study.

Species/Subspecies	Length (bp)					GC content (%)	Number of genes				
	Total	LSC	SSC	IR	PCGs		Total	CDS	tRNA	rRNA	Duplicated
*Dioscorea banzhuana*	153,989	84,004	18,959	25,513	79,071	37.20%	133	87	38	8	19
*Dioscorea biformifolia*	154,149	84,145	18,856	25,574	78,729	37.20%	133	87	38	8	19
*Dioscorea collettii*	153,746	83,903	18,657	25,593	78,867	37.20%	133	87	38	8	19
*Dioscorea deltoidea*	153,947	83,969	18,920	25,529	78,771	37.20%	133	87	38	8	19
*Dioscorea futschauensis*	153,948	83,981	18,909	25,529	78,759	37.20%	133	87	38	8	19
*Dioscorea gracillima*	153,996	83,970	18,908	25,559	78,777	37.20%	133	87	38	8	19
*Dioscorea nipponica*	153,885	83,950	18,919	25,508	78,780	37.20%	133	87	38	8	19
*Dioscorea nipponica* subsp. *rosthornii*	153,916	83,981	18,919	25,508	78,780	37.20%	133	87	38	8	19
*Dioscorea spongiosa*	153,947	83,969	18,920	25,529	78,771	37.20%	133	87	38	8	19
*Dioscorea tokoro*	153,946	83,968	18,920	25,529	78,765	37.20%	133	87	38	8	19
*Dioscorea villosa*	153,974	83,920	18,902	25,576	78,720	37.20%	133	87	38	8	19
*Dioscorea zingiberensis*	153,691	83,129	18,918	25,822	78,867	37.20%	133	87	38	8	19

### 2.5 Development of polymorphic nuclear SSRs

To develop polymorphic nuclear SSRs in sect. *Stenophora*, low-coverage whole genome sequence reads of these 11 species/subspecies were aligned to the reference genome sequence of D. *zingiberensis* (Cheng et al., 2021) to remove mitochondria and plastome reads, using BWA-MEM v.0.7.17 (Li, 2013). Aligned files were then sorted using SAMtools v.1.9 (Li et al., 2009). The resultant Binary Alignment/Map (BAM) data (only containing nuclear reads) were *de novo* assembled into scaffolds using a de Bruijn graph-based assembly program, SOAPdenovo v.1.0.4 (Xie et al., 2014). Based on these nuclear scaffolds, the potential polymorphic nuclear SSRs were identified using CandiSSR (Xia et al., 2016), with default parameters.

### 2.6 Phylogenetic analyses

Phylogenetic relationships among the 12 species/subspecies of sect. *Stenophora* (**Table 1**) were inferred based on two datasets: (1) whole plastome sequences and (2) 80 shared protein coding genes, taking *D. aspersa* (NC_039807) and *D. alata* (NC_039707) as outgroups. For the latter dataset, three partitioning scenarios: (1) unpartitioned scenarios; (2) partitioned by each gene and intergenic region; and (3) partitioned by codon position were employed. Both whole plastome sequences and protein coding sequences were aligned using the MAFFT v.7 plugin (Katoh and Standley, 2013) in Geneious Prime^®^ 2022.0.1. The best nucleotide substitution model was determined by the Akaike Information Criterion (AIC) in jModelTest v2.1.4 (Darriba et al., 2012), and the GTR + G substitution model was selected for both datasets. Maximum likelihood (ML) analyses were performed using RAxML v.8.2.12 (Stamatakis, 2014) available in the CIPRES Science Gateway v.3.3 (http://www.phylo.org/portal2/). Clade support values were estimated by 1000 bootstrap replicates. Bayesian inference (BI) analyses were conducted on MrBayes v.3.2.7 (Ronquist et al., 2012), which consists of two independent runs of 1 × 10^6^ generations, with four independent Markov chain Monte Carlo (MCMC) chains (i.e., one cold and three heated) each, and a sampling frequency of 1000 generations. The first 200 trees were discarded as ‘burn-in’, and the remaining trees were used to construct a majority-rule consensus tree and estimate posterior probabilities (PPs).

## 3 Results and discussion

### 3.1 Plastome characteristics

**Figure 1:**
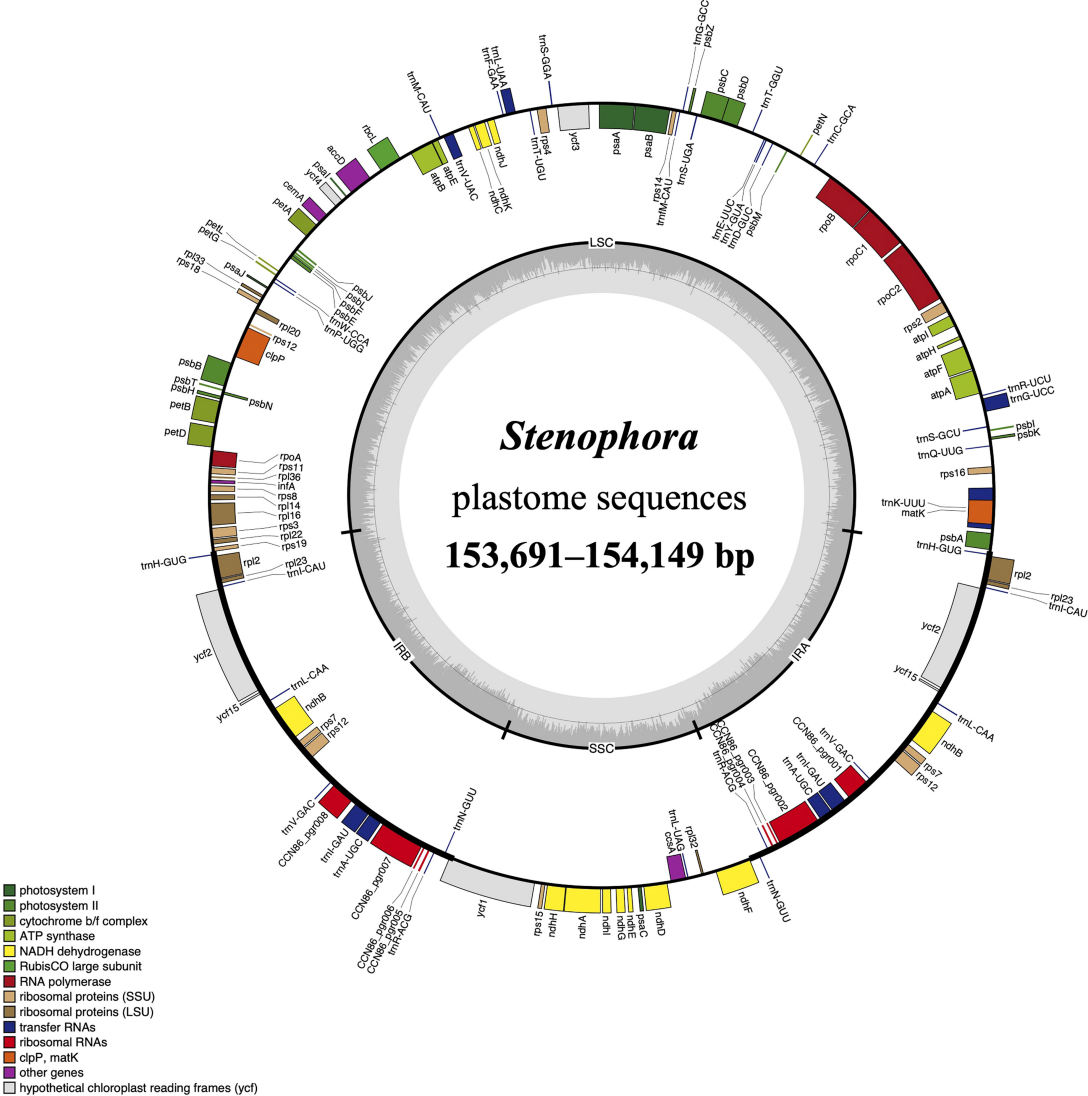
The plastome map of *Stenophora* species/subspecies. Thick lines on the outer complete circle identify the inverted repeat regions (IRa and IRb). Genes shown on the outside of the circle are transcribed clockwise, while genes inside are transcribed counter-clockwise. Genes are color coded according to their functional groups. GC/AT content is displayed by darker/lighter grey bars in the inner ring.

The whole length of these *Stenophora* plastomes ranged from 153,691 bp (*D. zingiberensis*) to 154,149 bp (*D. biformifolia*) (**Figure 1**; **Table 1**). All these plastomes shared the typical quadripartite structure of angiosperm plastomes, with a pair of IR regions (25,508–25,822 bp) separated by the LSC (83,129–84,145 bp) and SSC (18,657–18,959 bp) regions. The length variation of *Dioscorea* plastomes is a very common phenomenon (Zhao et al., 2018; Xia et al., 2019), which is often caused by the expansion and contraction of the IR regions (see details below). These *Stenophora* plastomes have the same overall GC content (37.20%), higher than that in LSC (35.00–35.10%) and SSC (31.2%) regions, but lower than that in IR regions (43.29–43.0%) (**Table 1**), possibly influenced by the high GC content (55.3%) of the four ribosomal RNA (rRNA) sequences.

All these *Stenophora* plastomes encoded the same 114 unique genes, including 80 protein-coding genes (PCGs), 30 transfer RNA (tRNA) genes, and four rRNA genes. Nineteen unique genes (seven PCGs, eight tRNA genes, and all four rRNA) were duplicated in the IRs, giving a total of 133 genes (**Figure 1**; **Table S1**). Among these unique genes, nine PCGs (i.e., *atpF*, *petB*, *petD*, *ndhA*, *ndhB*, *rpoC1*, *rpl2*, *rpl16*, and *rps16*) and six tRNAs (*trnK*-UUU, *trnG*-UCC, *trnL*-UAA, *trnV*-UAC, *trnI*-GAU and *trnA*-UGC) possessed a single intron, while three PCGs (*ycf3*, *rps12* and *clpP*) contained two introns (**Figure 1**; **Table S1**). The *rps12* gene consists of three exons that were trans-spliced together: exon 1 was located in the LSC region, while exons 2 and 3 were proximal and located in the IR regions (**Figure 1**; **Table S1**). Furthermore, all *Stenophora* plastomes reported in this study harbored the complete *rps16* gene, contrary to previous studies indicating the entire loss of *rps16* gene in several clades of *Dioscorea* (Jansen et al., 2007; Lu et al., 2023). To further improve our understanding of *rps16* gene evolution in *Dioscorea*, a ML phylogenetic tree was reconstructed (with the same method above) based on whole plastome sequences of 42 *Dioscorea* species, using *Trichopus zeylanicus* and *Tacca leontopetaloides* as outgroups. Phylogenetic result showed that *rps16* gene was lost in all other *Dioscorea* clades except *Stenophora*, suggesting a single loss of this gene within *Dioscorea* (**Figure S1**). Since *Stenophora* and the rest of the genus diverged about 48.3 (47.6–49.1) million years ago (Mya) (Viruel et al., 2016), the gene loss mentioned above may have occurred in sync with this divergence event, implying that the loss of *rps16* gene may have occurred about 48.3 Mya.

### 3.2 Comparative plastome analyses of *Stenophora*

**Figure 2:**
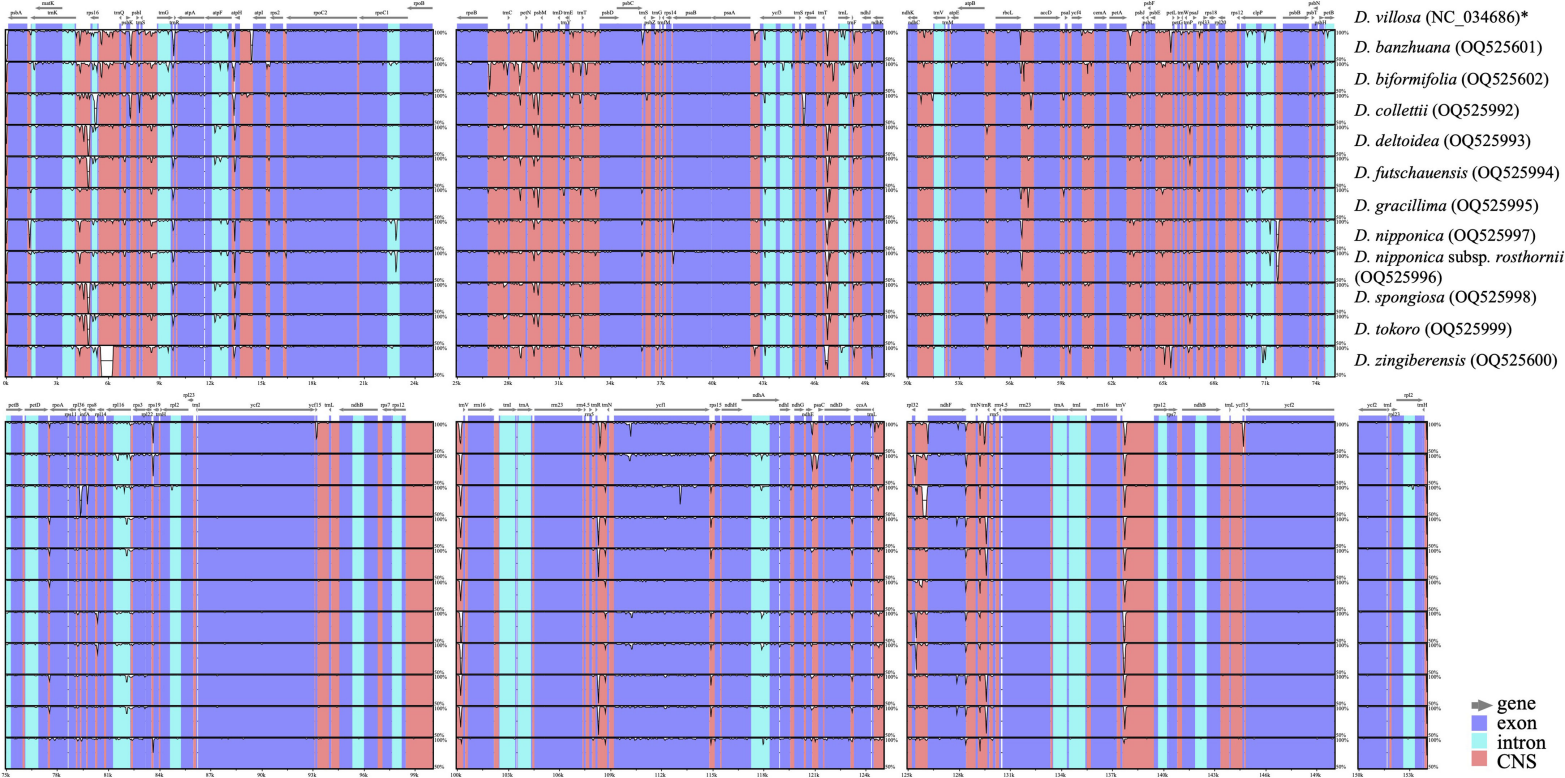
Sequence identity plots among *Stenophora* plastomes, with *D. villosa* (NC_034686) as a reference. Annotated genes are shown along the top. Gray arrows above the alignment indicates genes with their orientation. The vertical scale represents the percent identity between 50% and 100%. Genome regions are color coded as exon, intron, and conserved non-coding sequences (CNS). * previously published plastome sequence.

Comprehensive comparison of 12 *Stenophora* plastomes revealed a high degree of overall sequence similarity and collinearity within this section (**Figure 2**). Similar to previous monocot plastome studies (e.g., Asaf et al., 2017; Lu et al., 2017; Lu et al., 2021; Lu et al., 2022), our mVISTA analysis demonstrated that IRs exhibited a lower level of sequence divergence compared with LSC and SSC regions (**Figure 2**). This could be attributed to copy correction between IR sequences by gene conversion, and the abundance of conserved rRNA genes in the IRs (Khakhlova and Bock, 2006). In addition, the protein-coding regions were found to be more conserved than non-coding regions (including intergenic spacers and introns), which were likely to be subject to natural selection (Shaw et al., 2007; Lu et al., 2022).

**Figure 3:**
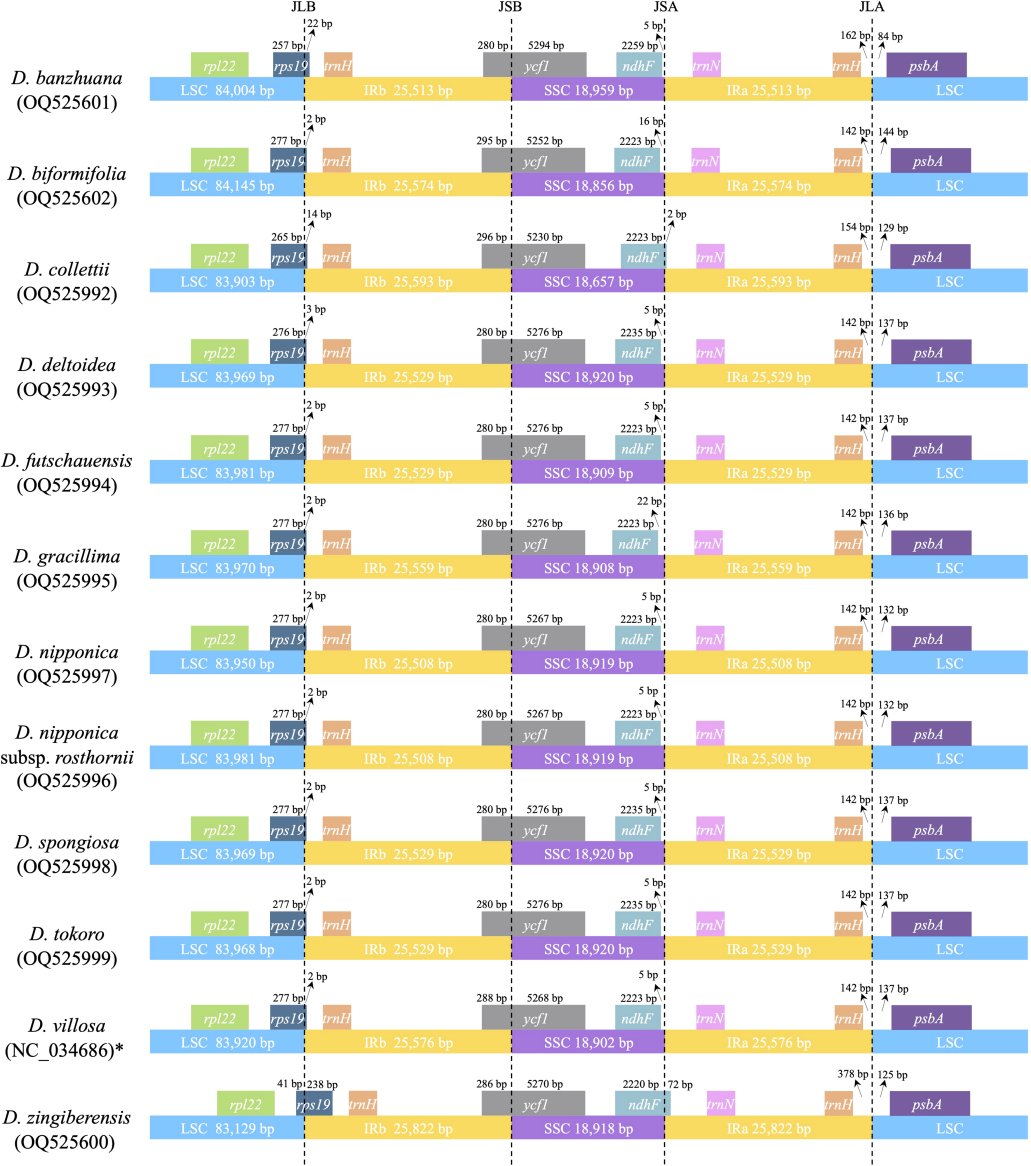
Comparison of IR/SC junctions among 12 *Stenophora* plastomes. * previously published plastome sequence.

Despite the similarity of plastome sequences, and the conservation of gene content and linear order of genes, the 12 *Stenophora* plastomes exhibited obvious differences at the IR/SC borders (**Figure 3**). For example, the *ndhF* gene crossed the SSC/IRa border in *D. collettii* and *D. zingiberensis*, while it was completely included in the SSC region in the other 10 species (**Figure 3**). The IRb region extended 238 bp into the *rps19* gene in *D. zingiberensis*, much deeper than those extended into all other species (2–22 bp), and further extended 280–296 bp into the *ycf1* gene. IR expansion into *rps19* gene has also been observed in other sections in the genus *Dioscorea*, e.g., *Opsophyton*, *Testudinaria*, *Enantiophyllum* (Zhao et al., 2018; Lu et al., 2023), suggesting that this phenomenon may be an ancestral symplesiomorphy of the genus *Dioscorea*. In addition, the *trnH* gene was totally located within the IR region and duplicated in all these species, 142–378 bp away from its proximal IR/SC border.

### 3.3 Plastome-derived hotspot regions and SSRs for *Stenophora*

**Figure 4:**
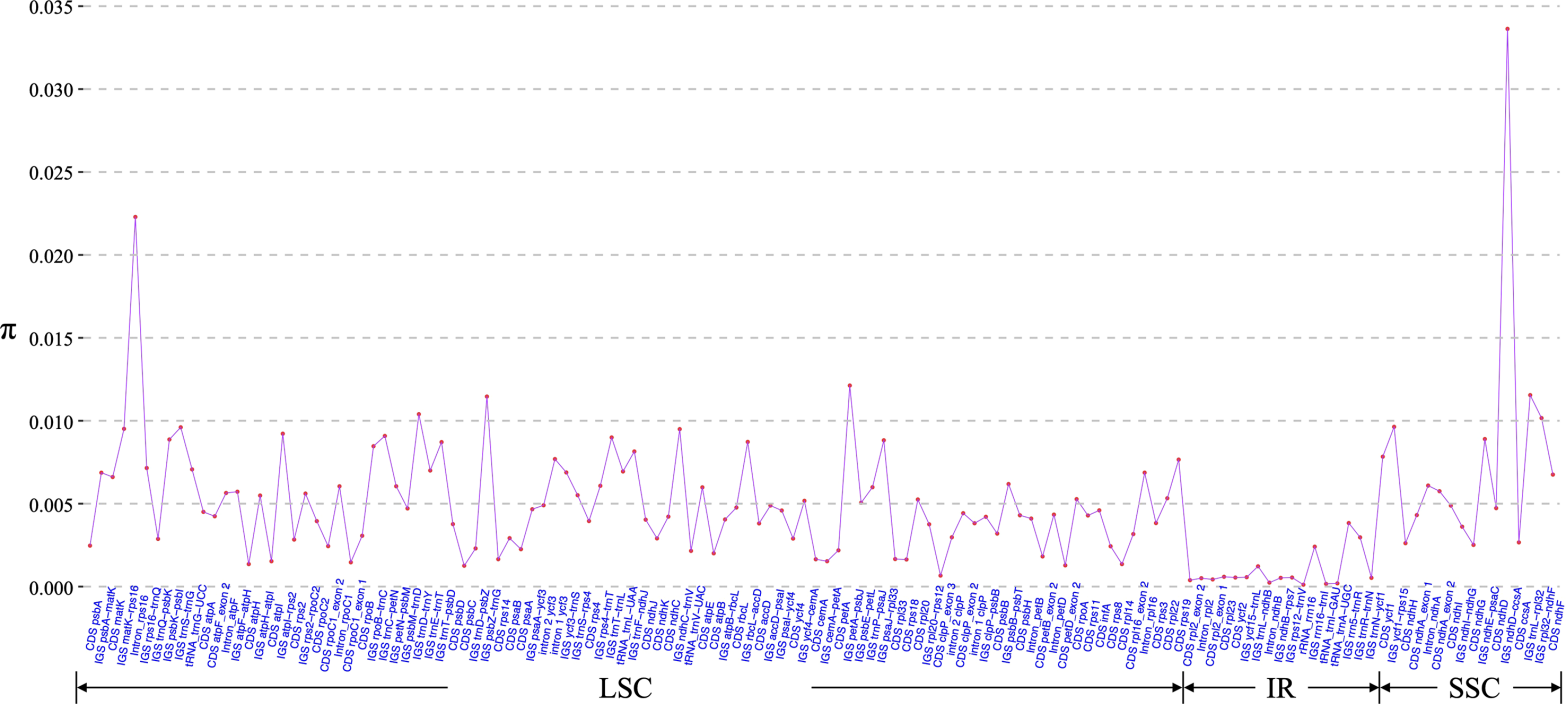
Nucleotide variability (π) values of 130 regions (58 CDS, 53 IGS, 13 introns, five tRNAs and one rRNA) extracted from the alignment matrix of 12 *Stenophora* plastome sequences.

Morphology-based species identification in sect. *Stenophora* has always been difficult, because it is challenging to find clear gaps of morphological variations among closely related species (Kawabe et al., 1997; Wilkin et al., 2005; Gao et al., 2008). In this case, barcoding has been performed for this section using nuclear gene phosphoglucose isomerase (PGI) and plastid DNA (*matK*, *rbcL* and *trnL-F*) regions (Kawabe et al., 1997; Gao et al., 2008). However, these markers are today considered intermediately variable regions (Shaw et al., 2014), and always showed low species discriminatory power and poor phylogenetic resolution (Gao et al., 2008; Noda et al., 2020). Therefore, we here used these plastome sequences to develop novel genetic markers (hypervariable regions) for taxonomic and phylogenetic analysis of *Stenophora*. A total of 130 regions (58 CDS, 53 IGS, 13 introns, five tRNAs and one rRNA) was eventually extracted to calculate the nucleotide diversity, and the π values ranged from 0.01% (*rrn16*) to 3.36% (*ndhD–ccsA*) (**Figure 4**). Six IGS regions (i.e., *ndhD-ccsA*, *petA-psbJ*, *trnL-rpl32*, *psbZ-trnG*, *trnD-trnY* and *rpl32-ndhF*), and *rps16* intron sequence were the top seven highly variable regions (π > 1.00%) (**Figure 4**), which could be served as section-specific molecular markers for future identification, conservation and utilization of *Stenophora* species.

**Figure 5:**
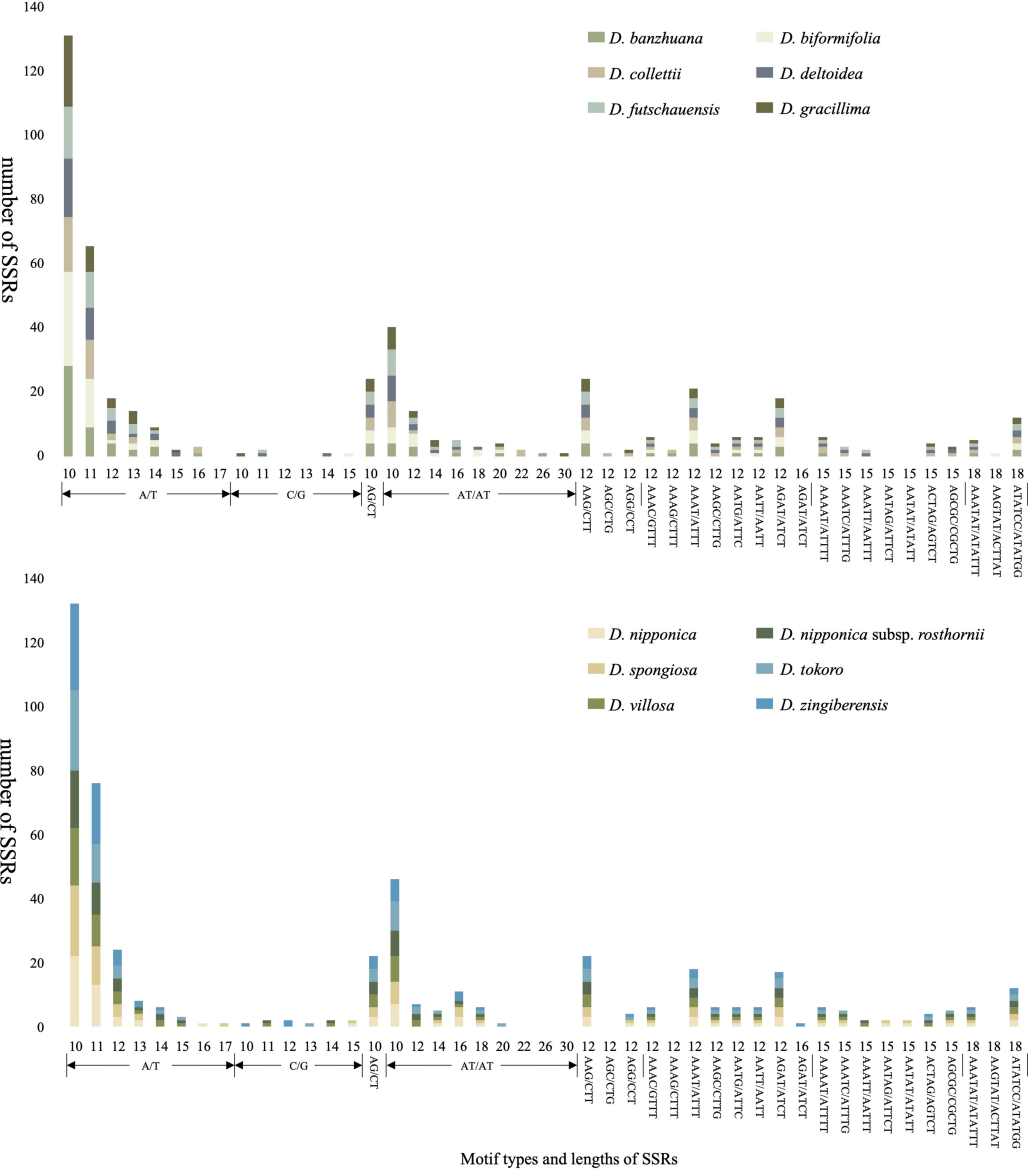
Plastome-derived SSRs in the 12 *Stenophora* species/subspecies.

Plastome-derived SSRs (chloroplast SSRs, cpSSRs) are scattered in the plastomes across different plant taxa, and have been widely used in population genetic studies and breeding programs (Jiménez, 2010; Chmielewski et al., 2015; Hazra et al., 2021; Ping et al., 2021). In this study, the MISA analysis identified a total of 960 SSRs across the 12 *Stenophora* plastomes. The number of SSRs for each plastome ranged from 74 (*D. futschauensis*) to 93 (*D. zingiberensis*). Mononucleotide repeats were predominant, with numbers ranging from 35 (*D. collettii*) to 50 (*D. biformifolia*), followed by dinucleotides ranging from 14 (*D. banzhuana*) to 18 (*D. villosa*), and tetranucleotides (10 in *D. collettii* and 9 in the other 11 plastomes), while trinucleotides (4–6 per plastome), pentanucleotides (0–5 per plastome) and hexanucleotides (3 per plastome) were relatively few in *Stenophora* plastomes (**Figure 5**; **Table S2**). The most common motifs were A/T and AT/TA for mono- and dinucleotides, accounting for 46.75%–68.76% and 17.20%–22.97% of the total SSRs in *Stenophora* plastomes, respectively, which may lead to the AT richness of the *Stenophora* plastomes (**Figure 5**; **Table S2**). These results were also consistent with the previous findings that plastome-based SSRs are largely composed of short polyadenine (polyA) and polythymine (polyT) repeats, while rarely contained tandem guanine (G) and cytosine (C) repeats (Kuang et al., 2011; Lu et al., 2022). In addition, several potential species-specific SSRs were identified in the present study. For example, AGC/CTG and AAGTAT/ACTTAT were only observed in *D. collettii* and *D. biformifolia*, respectively, but not appeared in the other 10 species/subspecies. Both AATAG/ATTCT and AATAT/ATATT were only presented in *D. nipponica* and its subspecies, while absence in other 10 species (**Figure 5**; **Table S2**). Clearly, these SSRs could be developed as effective molecular markers for species identification.

### 3.4 Polymorphic nuclear SSRs of *Stenophora*

Unlike plastome-derived SSRs with a certain degree of conservatism and usually uniparental inheritance, nuclear SSR markers are co-dominant and generally highly polymorphic, thus can complement plastome-derived SSR analysis in plants (Aecyo et al., 2021). In this study, based on the multiple assembled nuclear scaffolds of 11 newly sequenced *Stenophora* species/subspecies, a total of 12 polymorphic nSSRs (including six dinucleotides and six trinucleotides) were determined within this section by using CandiSSR (**Table S3**). Among these polymorphic nSSRs, nSSR_7 could divide these *Stenophora* species/subspecies into five groups, while four nSSRs (i.e., nSSR_1, nSSR_2, nSSR_5, nSSR_6) and seven nSSRs (i.e., nSSR_3, nSSR_4, nSSR_8, nSSR_9, nSSR_10, nSSR_11, nSSR_12) could divide them into four and three groups, respectively (**Table S3**). Apparently, these polymorphic nSSRs would be useful for species identification and conservation of this section, especially in the population genetic context.

### 3.5 Phylogenetic relationships within *Stenophora*

**Figure 6:**
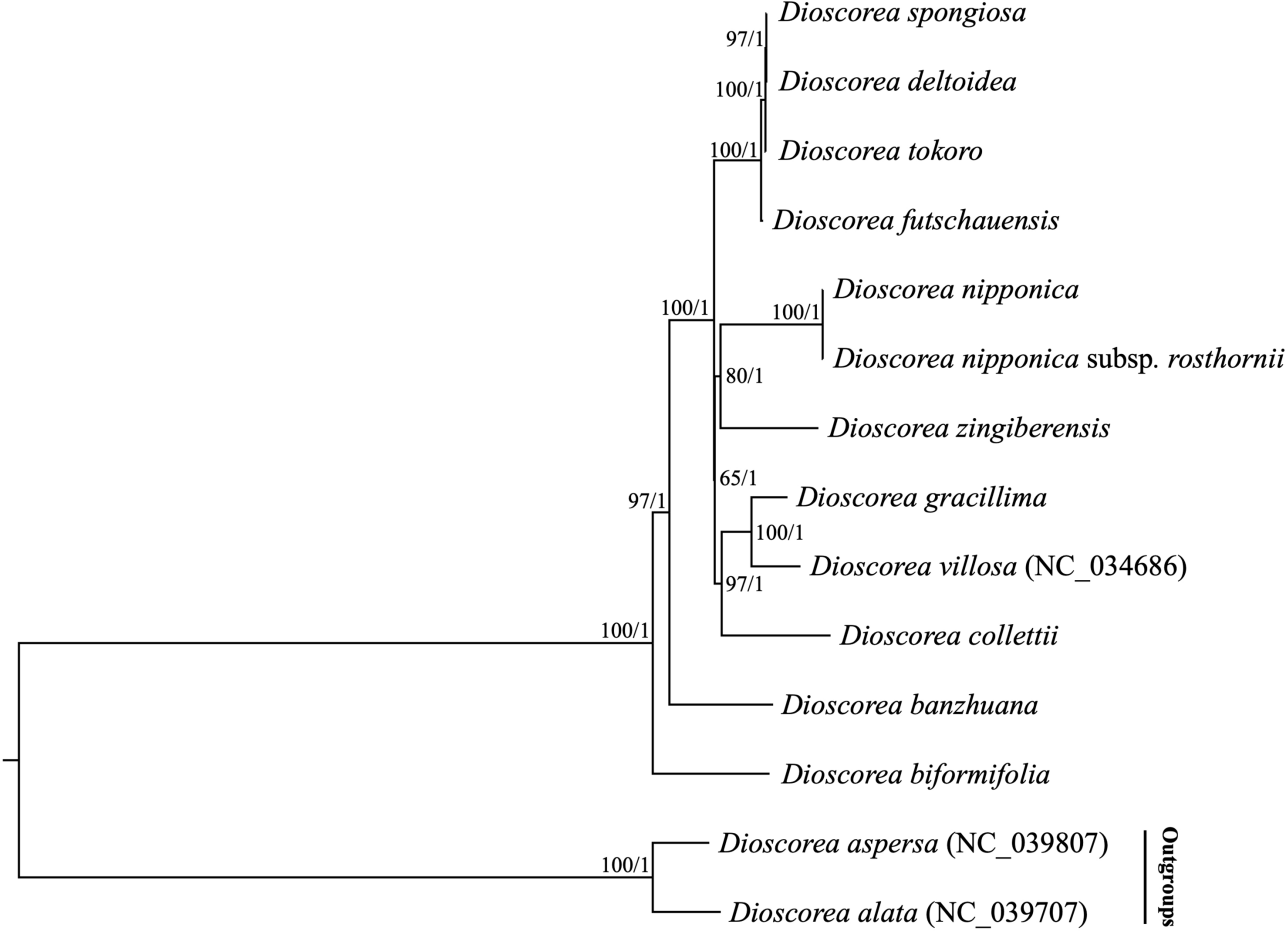
Phylogenetic relationships of 12 *Stenophora* species/subspecies inferred from Maximum likelihood (ML) and Bayesian inference (BI) methods, according to complete plastome sequences. Numbers above the lines represent ML bootstrap values and BI posterior probabilities. Phylogenetic trees based on 80 commonly present protein-coding genes with different partitioning scenarios are completely consistent with this topology.

Previous phylogenetic studies have laid an important foundation for the phylogeny and classification of *Stenophora* species, however the selected loci (e.g., *atpB*, *matK*, *rbcL* and *trnL-F*) unfortunately could not provide sufficient information for elucidating the phylogenetic and evolutionary relationships among them (Gao et al., 2008; Viruel et al., 2016; Noda et al., 2020). Recently, plastome sequences have been extensively used for phylogenetic analyses, especially in addressing unresolved relationship at low taxonomic levels (Carbonell-Caballero et al., 2015; Li et al., 2017). In this study, two datasets including the complete plastome sequences and 80 commonly present protein-coding genes of 12 *Stenophora* species/subspecies were used to perform phylogenetic analyses, with *D. aspersa* and *D. alata* as outgroups. Both ML and BI analyses of these two datasets (including different partitioning scenarios on protein-coding genes) yielded the same topology, with moderate to high bootstrap support values (BS = 65–100) and maximal posterior probability support values (PP = 1.0) at each node (**Figure 6**). The topology strongly supported *D. biformifolia* and *D. banzhuana* constituted the successive sister species to the rest. The remaining 10 sampled species/subspecies within this section could be further divided into three clades. Clade I contained four species, in which *D. futschauensis* was sister to the clade of *D. tokoro* + (*D. deltoidea* + *D. spongiosa*). Clade II, i.e., (*D. nipponica* + *D. nipponica* subsp. *rosthornii*) + *D. zingiberensis*, and clade III, i.e., (*D. gracillima* + *D. villosa*) + *D. collettii* were sister to each other, and jointly sister to Clade I (**Figure 6**). Contrary to previous hypothesis that *D. nipponica* ssp. *rosthornii* was not related to *D. nipponica* (Gao et al., 2008), our study strongly supported the monophyly of *D. nipponica* and *D. nipponica* ssp. *rosthornii*, which was consistent to the taxonomic treatments of this species in Flora of China (Ting et al., 2000).

Although our analyses have demonstrated the power of plastome phylogenetics to improve the resolutions of phylogenetic relationships in sect. *Stenophora*, this study was conducted based on insufficient taxa sampling, thus could not establish a complete picture of phylogenetic relationships within this section. Also considering that hybridization and polyploidization has been reported within this section (Qin and Zhang, 1985), plastome data could not accurately capture hybridization and polyploidization events, as plastome is usually uniparentally inherited, and acts as a linked single locus (Birky, 1995; Stull et al., 2015). Thus, moving beyond the plastomes and analyzing multilocus nuclear DNA sequence data with more extensive sampling is necessary in the future, to explore the phylogenetic and biogeographic hypotheses of sect. *Stenophora*.

## 4 Conclusions

In this study, we first assembled and annotated the complete plastomes of 11 *D*. sect. *Stenophora* species/subspecies, based on low-coverage whole genome sequencing data. Together with previously published plastome sequence of *D. villosa*, we then provided comparative plastome analyses within this section. All sampled *Stenophora* plastomes (153,691–154,149 bp) shared the same gene content, gene order and GC content. The *rps16* gene was lost in all other *Dioscorea* clades except *Stenophora*, which may have occurred about 48.3 Mya. A total of 960 plastome-derived SSRs and seven plastomic mutational hotspots were identified in *Stenophora*. Besides, we also successfully developed 12 polymorphic nuclear SSRs within this section, based on multiple assembled nuclear scaffolds. Phylogenetic analyses strongly supported that *D. biformifolia* and *D. banzhuana* constituted the successive sister species to the rest, which can be furtherly divided into three clades. Overall, the data obtained here will not only contribute to our understanding of plastome evolution of *Stenophora*, but also aid in the conservation and utilization of their genetic resources.

## Data availability statement

All data generated in this study has been publicly available.

## Author contributions

R-SL conceived the ideas. KH, X-QS and MC contributed to the sampling. X-QS and MC performed the experiment and analyzed the data. The manuscript was written by KH and R-SL. All authors contributed to the article and approved the submitted version.

## Supplementary material

Supplementary Figure 1 Phylogenetic relationships among *Dioscorea* based on complete plastome sequences. The loss of *rps16* gene is indicated on the branches of the tree.
