# Supplementary figures and images for: Low-coverage whole genome sequencing of eleven species/subspecies in Dioscorea sect. Stenophora (Dioscoreaceae): comparative plastome analyses, molecular markers development and phylogenetic inference
Source: Front Plant Sci. 2023 Jun 6;14:1196176. doi: 10.3389/fpls.2023.1196176 (PMC10281252; doi:10.3389/fpls.2023.1196176)

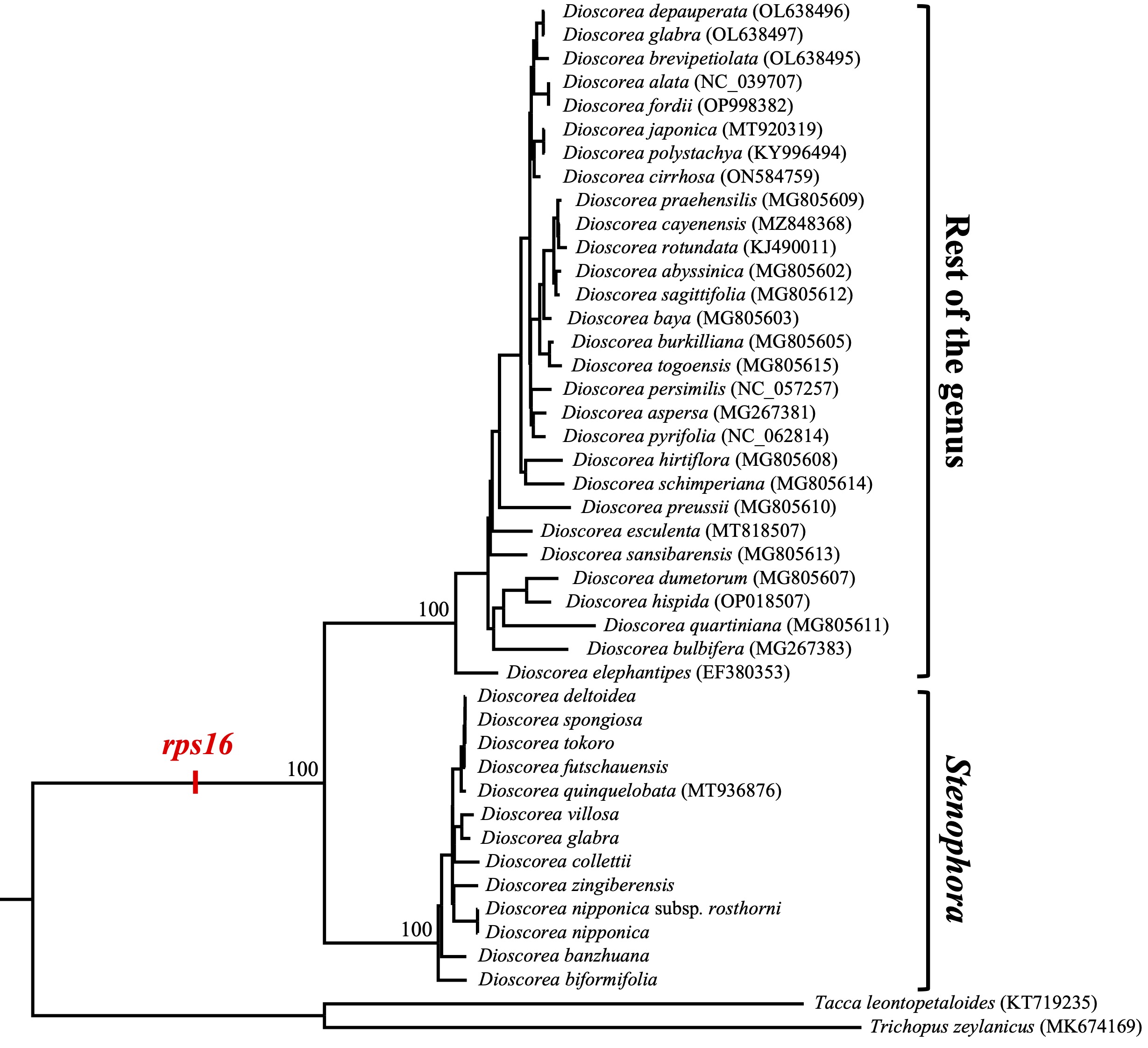

Supplement: Supplementary Figure 1 — Phylogenetic relationships among Dioscorea based on complete plastome sequences. The loss of rps16 gene is indicated on the branches of the tree. [file Image_1.jpeg]
