# Supplementary material for: Low-coverage whole genome sequencing of eleven species/subspecies in Dioscorea sect. Stenophora (Dioscoreaceae): comparative plastome analyses, molecular markers development and phylogenetic inference
Source: Front Plant Sci. 2023 Jun 6;14:1196176. doi: 10.3389/fpls.2023.1196176 (PMC10281252; doi:10.3389/fpls.2023.1196176)
Supplement: Supplementary file 2 [file Table_1.docx]

**Table S1** Gene compositions in plastomes of *Dioscorea* sect*. Stenophora*

| Category of genes | Group of genes | Name of gene |
| --- | --- | --- |
| RNA genes | Ribosomal RNAs | *rrn16* (×2), *rrn23* (×2), *rrn4.5* (×2), *rrn5* (×2) |
|  | Transfer RNAs | *trnK*-UUU^a^, *trnQ*-UUG, *trnS*-GCU, *trnG*-UCC^a^, *trnR*-UCU, *trnC*-GCA, *trnD*-GUC, *trnY*-GUA, *trnE*-UUC, *trnT*-GGU, *trnS*-UGA, *trnG*-GCC, *trnfM*-CAU, *trnS*-GGA, *trnT*-UGU, *trnL*-UAA^a^, *trnF*-GAA, *trnV*-UAC^a^, *trnM*-CAU, *trnW*-CCA, *trnP*-UGG, *trnH*-GUG (×2), *trnI*-CAU (×2), *trnL*-CAA (×2), *trnV*-GAC (×2), *trnI*-GAU^a^ (×2), *trnA*-UGC^a^ (×2), *trnR*-ACG (×2), *trnN*-GUU (×2), *trnL*-UAG |
| Photosynthesis related genes | Rubisco | *rbcL* |
|  | Photosystem I | *psaB*, *psaA*, *psaI*, *psaJ*, *psaC* |
|  | Assembly/stability of photosystem I | *ycf3*^b^, *ycf4* |
|  | Photosystem II | *psbA*, *psbK*, *psbI*, *psbM*, *psbD*, *psbC*, *psbZ*, *psbJ*, *psbL*, *psbF*, *psbE*, *psbB*, *psbT*, *psbN*, *psbH* |
|  | ATP synthase | *atpA*, *atpF*^a^, *atpH*, *atpI*, *atpE*, *atpB* |
|  | Cytochrome b/f complex | *petN*, *petA*, *petL*, *petG*, *petB*^a^, *petD*^a^ |
|  | Cytochrome c synthesis | *ccsA* |
|  | NADH dehydrogenease | *ndhJ*, *ndhK*, *ndhC*, *ndhB*^a^ (×2), *ndhH*, *ndhA*^a^, *ndhI*, *ndhG*, *ndhE*, *ndhD*, *ndhF* |
| Transcription and translation related genes | Transcription | *rpoC2*, *rpoC1*^a^, *rpoB*, *rpoA* |
|  | Ribosomal proteins (large units) | *rpl33*, *rpl20*, *rpl36*, *rpl14*, *rpl16*^a^, *rpl22*, *rpl2*^a^ (×2), *rpl23* (×2), *rpl32* |
|  | Ribosomal proteins (small units) | *rps16^a^*, *rps2*, *rps14*, *rps4*, *rps18*, *rps12*^b^ (×2), *rps11*, *rps8*, *rps3*, *rps19*, *rps7* (×2), *rps15* |
|  | Translation initiation factor | *infA* |
| Other genes | RNA processing | *matK* |
|  | Fatty acid synthesis | *accD* |
|  | Caseinolytic protease proteolytic subunit | *clpP*^b^ |
|  | Carbon metabolism | *cemA* |
| Genes of unknown function | Conserved reading frame | *ycf2* (×2), *ycf15* (×2), *ycf1* |

^a^ indicates the genes containing a single intron; ^b^ indicates the genes containing two introns; (×2) indicates genes duplicated in the IR regions.
